# Small-Sized Interferometer with Fabry–Perot Resonators for Gravitational Wave Detection

**DOI:** 10.3390/s21051877

**Published:** 2021-03-08

**Authors:** Nikolai Petrov, Vladislav Pustovoit

**Affiliations:** Scientific and Technological Centre of Unique Instrumentation of the Russian Academy of Sciences, 15 Butlerova str., 117342 Moscow, Russia; vladpustovoit@gmail.com

**Keywords:** Mach–Zehnder interferometer, Fabry–Perot resonator, periodic diffraction structure, gravitational wave detection, spectral resolution, frequency shift

## Abstract

It is highly desirable to have a compact laser interferometer for detecting gravitational waves. Here, a small-sized tabletop laser interferometer with Fabry–Perot resonators consisting of two spatially distributed “mirrors” for detecting gravitational waves is proposed. It is shown that the spectral resolution of 10^−23^ cm^−1^ can be achieved at a distance between mirrors of only 1–3 m. The influence of light absorption in crystals on the limiting resolution of such resonators is also studied. A higher sensitivity of the interferometer to shorter-wave laser radiation is shown. A method for detecting gravitational waves is proposed based on the measurement of the correlation function of the radiation intensities of non-zero-order resonant modes from the two arms of the Mach–Zehnder interferometer.

## 1. Introduction

The idea of using laser interferometers to detect gravitational waves was first expressed in 1962 in Reference [1], where a new method for registering gravitational waves was proposed, based on the use of a laser interferometer. In 2015, these waves were detected experimentally by two detectors of the Laser interference gravitational wave Observatory (LIGO) in Hanford and Livingston (USA) [2,3]. A detailed analysis of the current state is given in the review [4]. 

It is known that the sensitivity of detectors for detecting gravitational waves (GWs) is determined by the reflectivity of mirrors. The minimum displacement of the body that can be registered is proportional to the reflectivity of mirrors in the gravitational wave detector (*x*_min_~(1 − *R*), where *R* is the mirror reflection coefficient) [5,6,7]. Thus, to increase the sensitivity of the detector, it is necessary to increase the reflection coefficient *R*. The main problems that limit the capabilities of detectors are quantum shot noise and thermal effects due to the absorption of optical laser radiation. Increasing the stored power in the Fabry–Perot resonator reduces quantum shot noise at high frequencies inversely proportional to the square root of the stored power. The Advanced LIGO interferometer is supposed to increase the stored power to 750 kW [8], which will allow achieving the necessary sensitivity of the measuring system. High power in the interferometer leads to several effects that interfere with system control. These are angular instabilities due to light pressure [9], parametric instabilities [10], and thermo-optical distortions due to optical power absorption [11]. Planned cryogenic gravitational wave detectors will require improved coatings with deformational thermal noise reduced by 25 times compared to Advanced LIGO. In Reference [12], a multi-layer structure is proposed as a new coating material for future detectors, which for the first time can simultaneously meet the strict requirements for optical absorption and thermal noise of the Einstein cryogenic telescope. However, the use of multi-layer coatings does not solve the problem of thermal noise. 

The problem of increasing the sensitivity of gravitational antennas leads to the need to manufacture mirrors with a very high reflection coefficient, or to further increase the interferometer arms, as it is supposed to do in the space version of the system (the LISO project). 

New features appear when using distributed resonator mirrors, which are three-dimensional diffraction gratings created in the volume of the crystal. As it was shown in Reference [13], super-narrow transparency lines of such a resonator appear near the Bragg frequency. The use of Fabry–Perot resonators with periodic structures as reflecting mirrors for detecting gravitational waves was considered in Reference [14]. It has been shown that when using such resonators, the sensitivity of the laser gravitational antenna can be significantly increased compared to conventional Fabry–Perot interferometers. The distance between the periodic structures was assumed to be 628 m, which is significantly less than the distance between the mirrors in the LIGO installation. Note that the sensitivity estimates of the proposed measurement method for changes in the interference pattern were made for a zero-order maximum. It is interesting to evaluate the sensitivity of the measurement method by shifting the maxima of resonant modes of non-zero order [15].

This paper shows the possibility of creating a laser interferometer with distributed mirrors for detecting gravitational waves in laboratory conditions. As reflecting mirrors forming the optical Fabry–Perot interferometer, it is proposed to use reflecting periodic structures that have an exponentially narrow bandwidth of laser radiation. It is shown that the spectral resolution of 10^−23^ cm^−1^ can be achieved at a distance between mirrors of only 1–3 m. The influence of light absorption in crystals on the limiting resolution of such resonators is also studied. A method is proposed based on measuring the correlation function of the intensity of non-zero-order resonant modes from the two arms of the Mach–Zehnder interferometer, whose sensitivity exceeds that of the LIGO system. 

## 2. Fabry–Perot Resonator with Periodical Structures as Reflecting Mirrors

**Figure 1:**
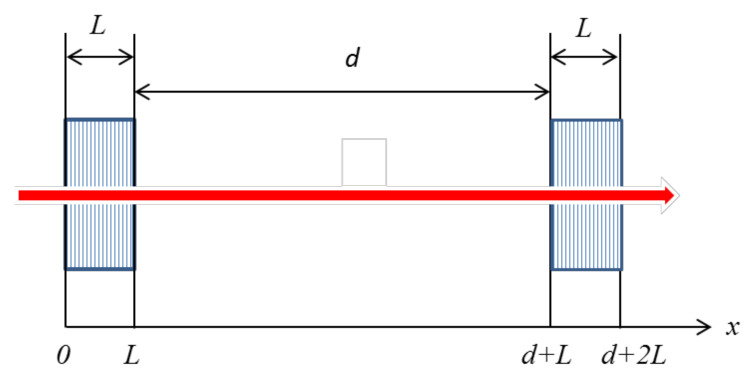
Fabry–Perot resonator with periodic structures as reflecting mirrors.

Consider a Fabry–Perot resonator formed by two periodic structures that are located at a distance *d* relative to each other (Figure 1). The radiation propagates from left to right. Our goal is to find the intensity of the radiation that came out of the resonator (or the hardware function of the resonator). 

The modulation of the dielectric constant of mirrors in a Fabry–Perot resonator is given by the expression

(1) $$\varepsilon (x)=\varepsilon_{0}+\Delta \varepsilon \cos(qx)$$

where $\varepsilon_{0}$ is the constant component of the dielectric constant, *q* is the wavevector of the periodic structure, Δε is the modulation amplitude of the dielectric constant, and $\Delta \varepsilon <<\varepsilon_{0}$.

The equations for coupled modes in a periodic structure (1) derived from Maxwell’s equations have the form [14,16]:

(2) $$\begin{array}{l} \frac{dE_{1}}{dx}=-ik_{0}\Delta \varepsilon (x)e^{i\Delta kx}E_{2}\\ \frac{dE_{2}}{dx}=ik_{0}\Delta \varepsilon (x)e^{-i\Delta kx}E_{1} \end{array}$$

where *E*_1_ is the amplitude of the incident wave, *E*_2_ is the amplitude of the reflected wave, $k_{0}=2\pi /\lambda$ is the wavenumber of the radiation, $\Delta k=2k_{0}-q$ is the wave detuning that determines the deviation from the Bragg synchronism condition. 

Equation (2) describe the propagation of light in the first (left) periodic structure (0≤x≤L). The equations describing the propagation of light in the right periodic structure in the region L+d≤x≤d+2L, in contrast to Equation (2), Δε will contain a phase factor $e^{i\varphi }$, where φ is the phase difference between the periodic structures, which occurs between the first and second periodic structures by extending a first periodic structure on the area L+d≤x≤d+2L.

Solutions of Equation (2) will be sought under the following boundary conditions:

(3) $$E_{1}(x=0)=E_{0},\;E_{2}(x=L)=e^{-ikd}E_{2}^{\prime }(x=L+d);\;E_{1}^{\prime }(x=L+d)=e^{ikd}E_{1}(x=L),\;E_{2}^{\prime }(x=2L+d)=0,$$

where the dash at the top indicates the fields of the light wave in the second periodic structure, *L* is the thickness of the mirror (crystal), and *d* is the distance between the mirrors. 

The amplitude of the diffracted wave and the reflection coefficient in the case of a medium with a constant value Δε along the length of the crystal is determined analytically [16]. The case, when the value Δε changes along with the crystal, is also of practical interest for controlling the hardware function of the resonator. However, in this case, Equation (2) can only be solved by numerical methods [13,17]. The influence of various apodization functions of Δε on the diffraction curves of reflection and transmission, taking into account light absorption, was studied in Reference [18]. It is shown in Reference [19] that the reflection and transmission coefficients are affected by the polarization of the incident radiation and changes in the geometric parameters and the concentration of dielectric inclusions in the medium. The solution of the boundary value problem (3) leads to the following expression for the amplitude of the wave exiting the resonator:

(4) $$t=\frac{E_{0}^{\prime }(2L+d)}{E_{0}(0)}=\frac{s^{2}\exp(ikd+i\Delta kd/2+3i\Delta kL/2)}{{\left(s\cdot ch(sL)-(\Delta k/2)sh(sL)\right)}^{2}+{\left|\Gamma \right|}^{2}sh^{2}(sL)\exp(-i\Delta kd/2-i\varphi )}$$

where $s={\left({\left|\Gamma \right|}^{2}-{\left(\Delta k/2\right)}^{2}\right)}^{1/2}$, $\Gamma =\Delta \varepsilon k_{0}$, φ is the phase difference and *d* is the distance between two periodic structures. 

The relative intensity of the transmitted radiation or the transmission coefficient is determined by the expression

(5) $$T={\left|t\right|}^{2}={\left|\frac{E_{0}^{\prime }(2L+d)}{E_{0}(0)}\right|}^{2}$$

**Figure 2:**
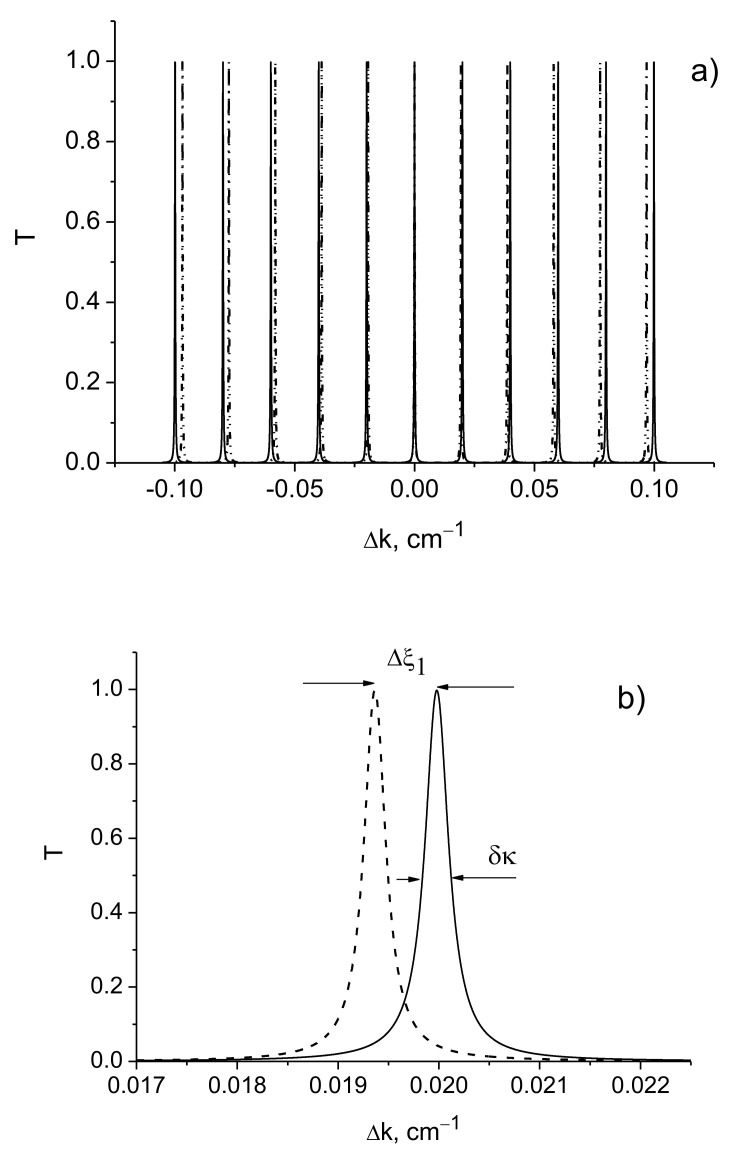

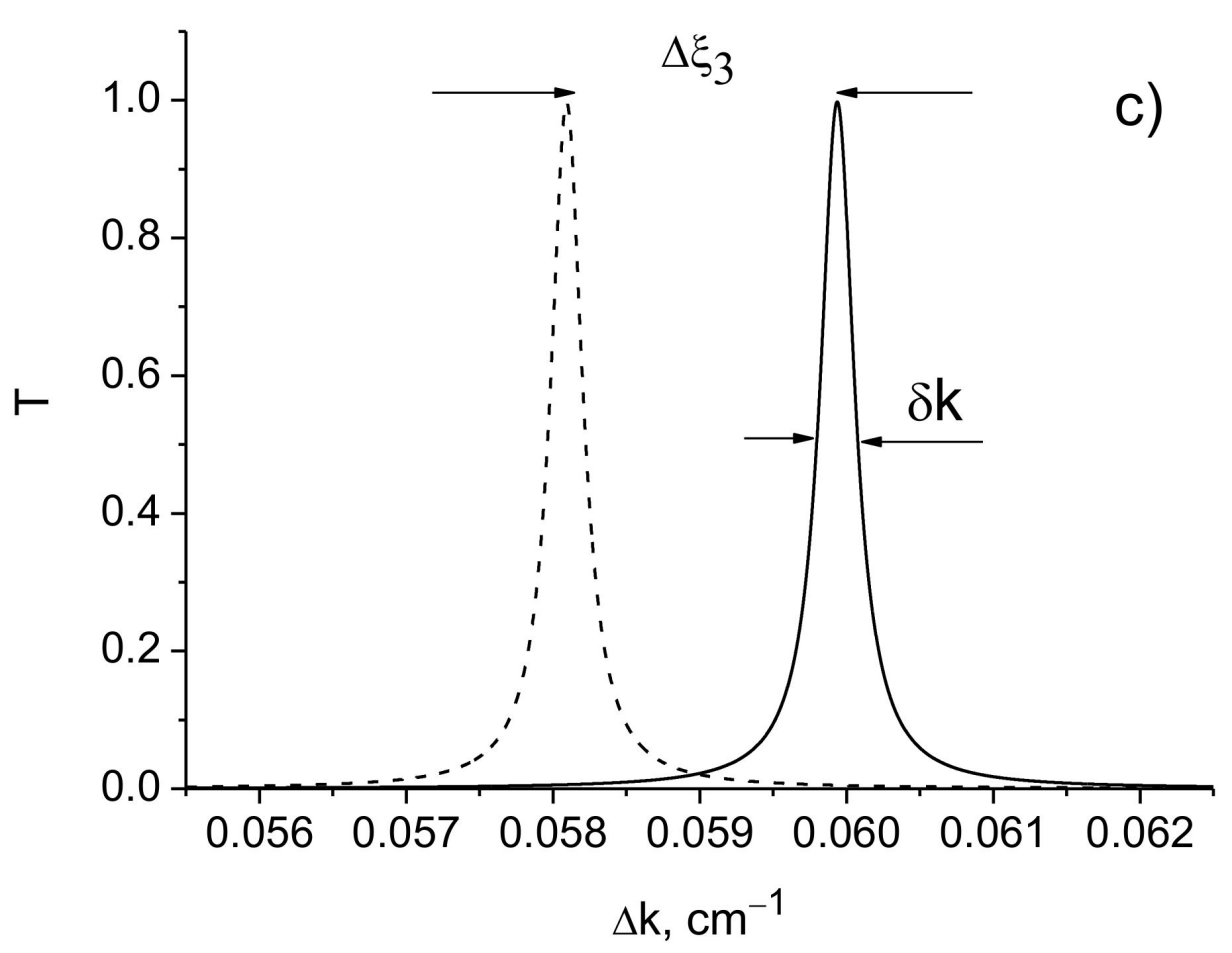
Transmittance as function of detuning Δk. (**a**) A set of peaks from the two arms of the Mach–Zehnder interferometer with different distances between the mirrors; (**b**) the peaks corresponding to the 1st order modes; (**c**) the peaks corresponding to the 3rd order modes. *d* = 100π cm, *L* = 0.75 cm, *λ* = 630 nm.

The general shape of the transmission function is shown in Figure 2. It is seen that the transmission spectrum consists of a set of maxima (Figure 2). The transmission maxima are observed when the Bragg resonance condition is fulfilled and their frequencies are determined by the distance between the mirrors *d*:

(6) $$\xi_{m}=\frac{2m\pi }{d},\;m=0,\pm 1,\pm 2,\ldots ,$$

where *m* is the resonance mode order.

When the phase shift in the harmonic profile of the refractive index distribution of the first and second mirrors is equal to φ=π and the exact synchronization condition is met, the main zero-order maximum occurs. Note that the zero order disappears for other phase values.

It follows from simulations (Figure 2) that the shift between the resonant modes from the two arms of the Mach–Zehnder interferometer increases with the order of the recorded mode. However, the line width (spectral resolution) of the modes remains constant at the same time. This indicates that the sensitivity of the interferometer increases with an increase in the order of the recorded resonant modes. 

The frequency distance Δζ between the transmission maximums is defined by the expression

(7) $$\Delta \zeta =\xi_{m+1}-\xi_{m}=\frac{2\pi }{d}$$

It can be seen from Figure 2 that a change in the distance between mirrors entails a change in the transmission spectrum of the interferometer: the distance between the maxima changes due to the frequency shift of the resonant modes of non-zero order with m≠0. Note that the shift of the peaks increases with the order of the mode. 

**Figure 3:**
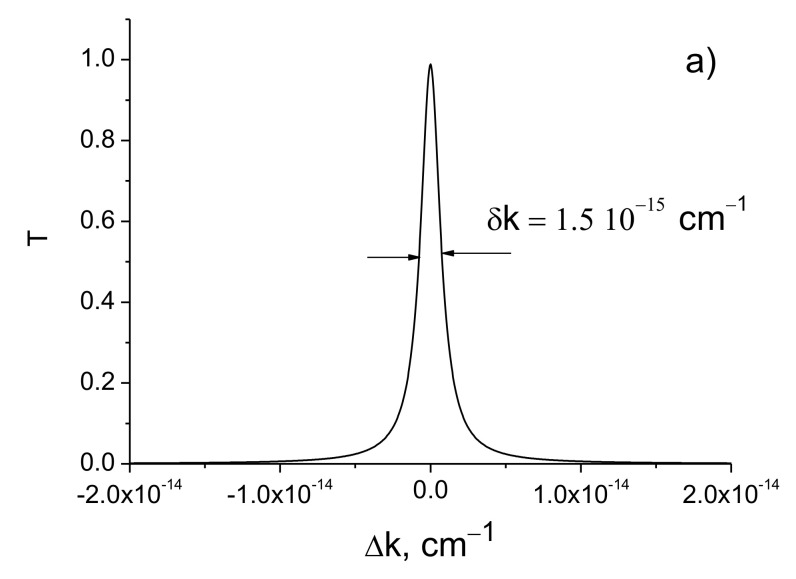

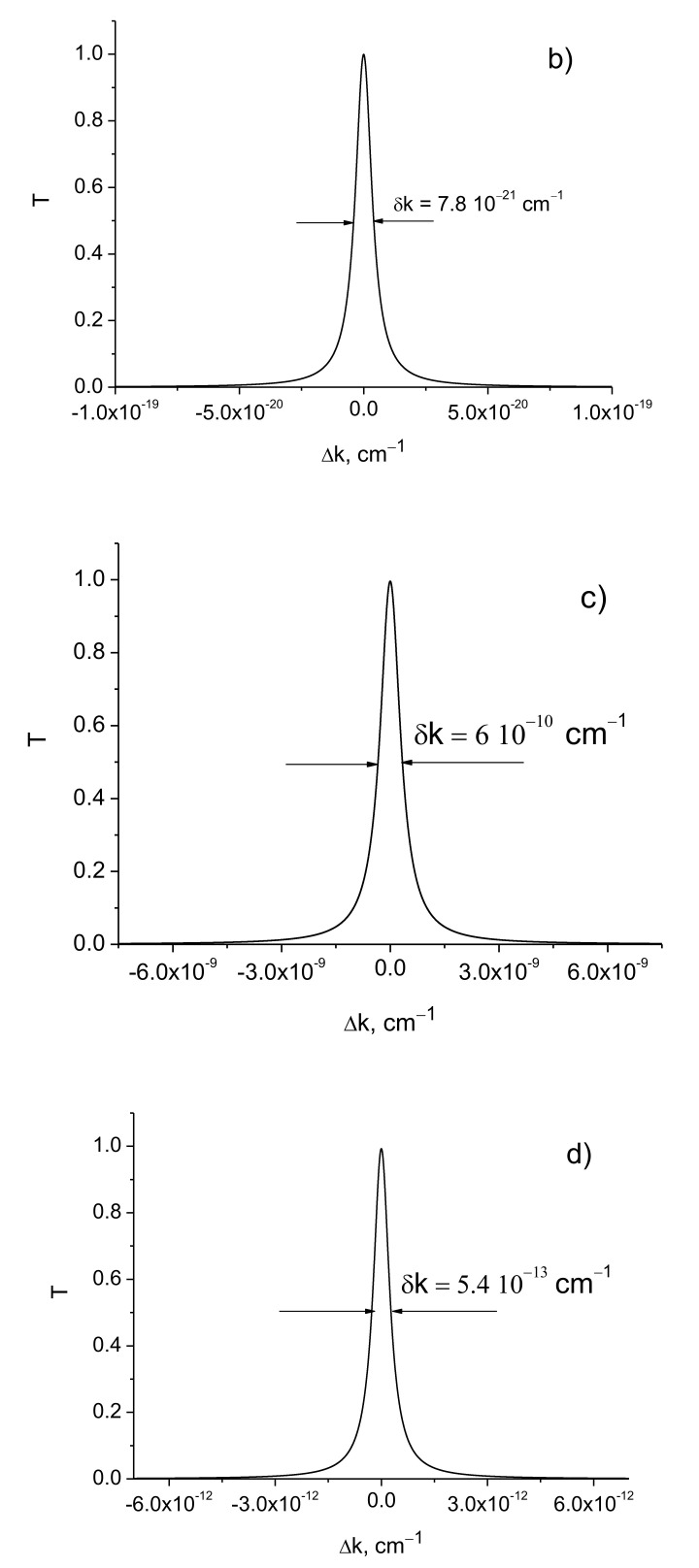
Transmission coefficients as function of the detuning of the resonator. *L* = 0.75 cm, *d* = 1 m. (**a**) $\Delta \varepsilon =2.1\cdot 10^{-4}$, *λ* = 630 nm; (**b**) $\Delta \varepsilon =2.9\cdot 10^{-4}$, *λ* = 630 nm; (**c**) $\Delta \varepsilon =2.1\cdot 10^{-4}$, *λ* = 1064 nm; (**d**) $\Delta \varepsilon =2.9\cdot 10^{-4}$, *λ* = 1064 nm.

Figure 3 shows the transmission curves for different values of the dielectric constant (permittivity) modulation amplitude Δε and radiation wavelength *λ*.

As follows from the simulations, the width of the transmission line decreases sharply with the increase of the amplitude of the refractive index modulation. The line width of the transmittance can reach the values of $\delta k≃10^{-23}$ cm^−1^ at $\Delta \varepsilon =3.3\cdot 10^{-4}$ and *d* = 1 m for the radiation wavelength 630 nm. This indicates that the spectral resolution of the filter is equal to

$$\delta \lambda /\lambda_{0}=\delta k/k_{0}=\frac{\lambda_{0}}{2\pi }\cdot 10^{-23}{\mathrm{cm}}^{-1}≃10^{-28}$$

Such a resolution of the filter corresponds to the quality factor of the resonator $Q\approx 10^{28}$. 

The spectral line widths δk increase significantly with the increase of the radiation wavelength (Figure 3c,d and Table 1). This suggests that the use of short-wave laser radiation is more preferable.

When φ=π, the hardware function near the zero maximum has a Lorentz form and can be represented as:

(8) $$T(L,\Gamma ,\Delta k,d)≃\frac{1}{1+{\left(\Delta k/\delta k\right)}^{2}}$$

where $\delta k≃\frac{4\exp(-2\Gamma L)}{d}$ is the width of the transmission curve determined at the half-height of the maximum, and ΓL>1. 

Of practical interest is the estimation of the effect of asymmetry caused by the difference in the thickness of the left and right periodic structures δL = *L*_1_ − *L*_2_. Modeling shows that such an asymmetry leads to a decrease in the amplitude of the transmission coefficient and a change in the width of the spectral line (Table 2). However, with existing manufacturing technologies, the effect of thickness errors on each mirror will be insignificant.

Imperfections in the manufacturing process, such as deviations from perfectly symmetrical periodic structures in Fabry–Perot resonators, can also reduce the sensitivity of the interferometer. One of the parameters is the difference in the modulation amplitudes of the dielectric constants of the left and right periodic structures $\delta \varepsilon =\Delta \varepsilon_{1}-\Delta \varepsilon_{2}$. It follows from the simulation that a 1% difference in the modulation amplitude will not lead to a significant change in the spectral line width and the transmission coefficient at moderate values of the modulation amplitude (Table 3). However, for the highest values of the modulation amplitude, this deviation cannot be ignored. Alignment of the incident beam wavefront with periodic structures is important for reducing Fresnel losses. To avoid this problem, laser beam expanders to shape a wavefront with a high degree of accuracy can be used. Currently, various types of divergence adjustable laser beam expanders are designed to compress the beam divergence angle by expanding the laser beam diameter, and they can also be used to adjust the divergence angle to compensate for the input beam divergence. 

**Figure 4:**
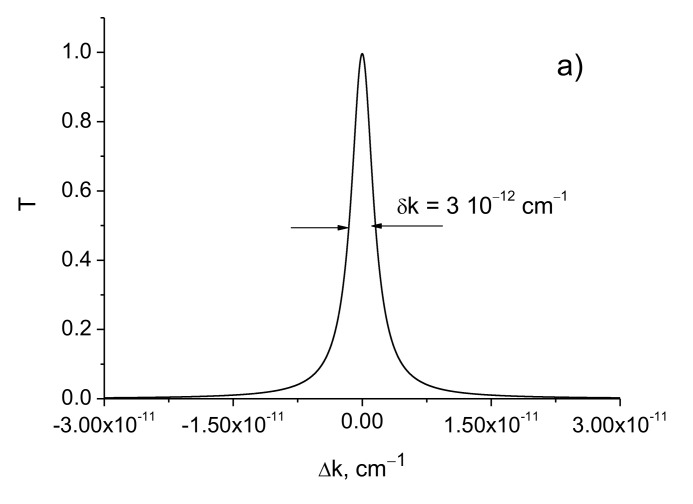

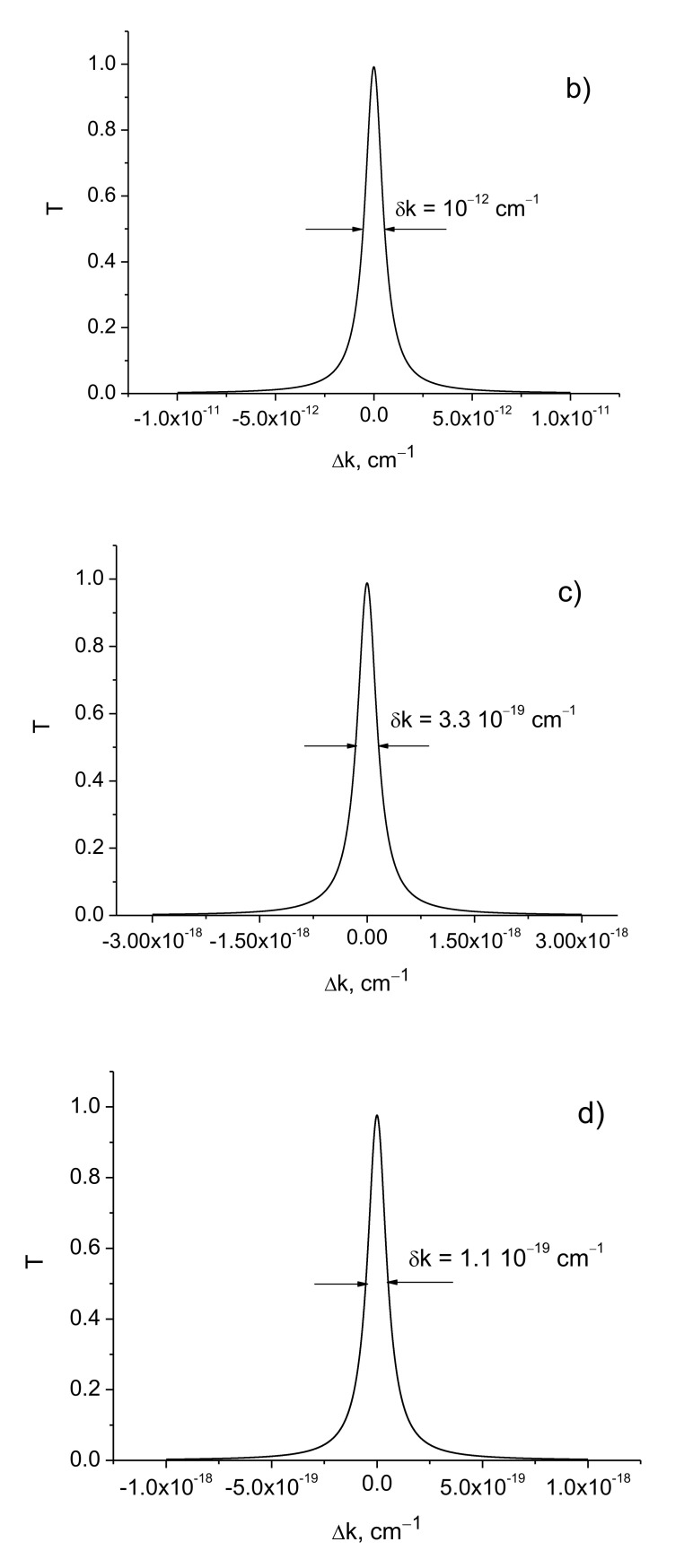
Transmission coefficients as function of the detuning of the resonator. *λ* = 630 nm, *L* = 0.40 cm. (**a**) $\Delta \varepsilon =3.0\cdot 10^{-4}$, *d* = 1 m; (**b**) $\Delta \varepsilon =3.0\cdot 10^{-4}$, *d* = 3 m; (**c**) $\Delta \varepsilon =5.0\cdot 10^{-4}$, *d* = 1 m; (**d**) $\Delta \varepsilon =5.0\cdot 10^{-4}$, *d* = 3 m.

The reduction of the thickness of the periodic structure can be performed with the preservation of the resolution while increasing the distance between the structures or the amplitude of the modulation of the permittivity. Figure 4 shows the transmission coefficients depending on the detuning at different values of the distance *d* between the mirrors and the amplitude of the dielectric constant modulation Δε.

It can be seen that the transmission line width decreases linearly with increasing distance *d*, while the dependence on the modulation amplitude of the permittivity is exponential (Figure 4 and Table 4). 

It is interesting to compare the sensitivity of the proposed method with the sensitivity of the measurement method in the LIGO installation, which uses a conventional Fabry–Perot interferometer with freely suspended mirrors. The mirror reflection coefficient achieved so far is *R* = 0.999994, and the distance between the mirrors is $d=4\cdot 10^{5}$ cm.

The expression for the hardware function of a conventional Fabry–Perot interferometer has the form [20]:

(9) $$T_{F-P}=\frac{1}{1+\frac{4R^{2}}{{\left(1-R^{2}\right)}^{2}}\sin^{2}\frac{n_{0}kd}{2}}≃\frac{1}{1+\frac{4R^{2}}{{\left(1-R^{2}\right)}^{2}}{\left(\frac{n_{0}d}{2}\right)}^{2}{\left(\Delta k\right)}^{2}},$$

where *R* is the reflection coefficient of the mirrors, *d* is the distance between mirrors, *n*_0_ is the refractive index of the medium between the mirrors, *n*_0_ = 1.

**Figure 5:**
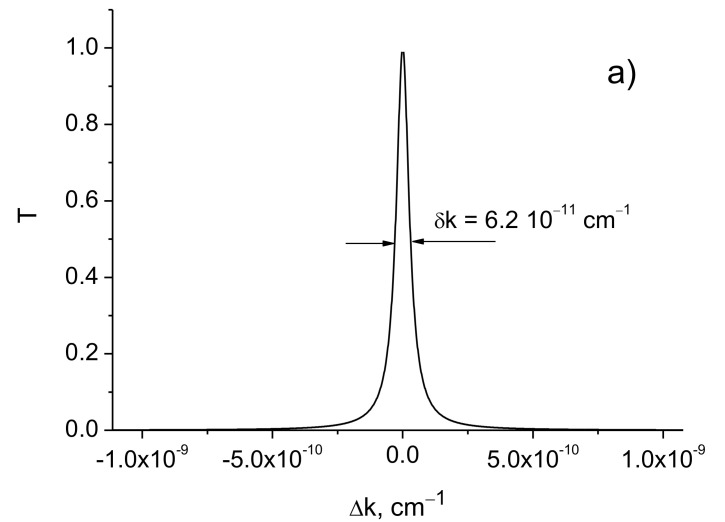

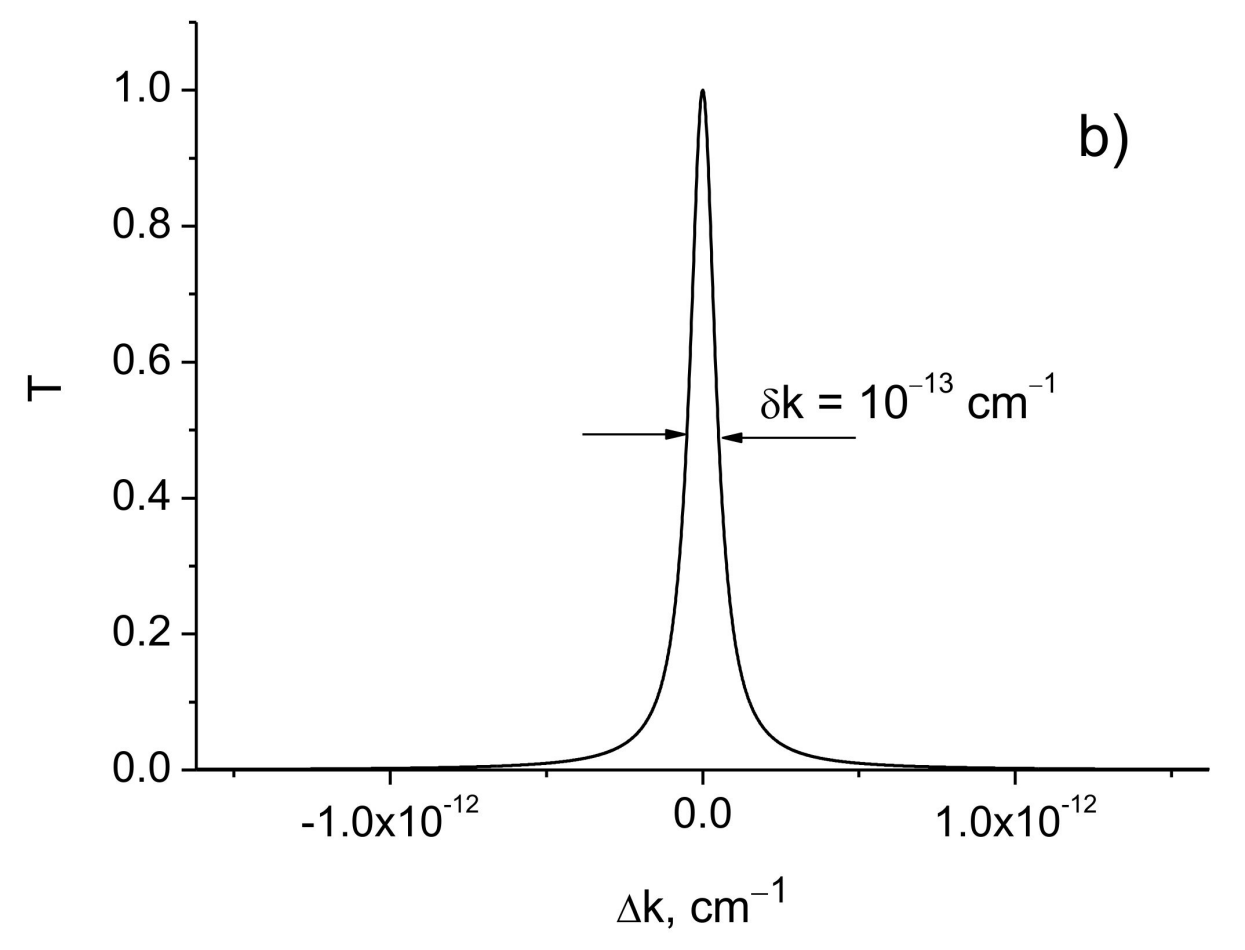
Transmission curves of the conventional *F-P* resonator: (**a**) 1 − *R* = 6 10^−6^; (**b**) 1 − *R* = 10^−8^. *d* = 4 km, *λ*_0_ = 630 nm.

Figure 5 shows the transmission curves calculated using the formula (9) for different values of the reflection coefficient *R*.

Table 5 shows the transmission curve widths obtained from (9) for the different reflection coefficients *R* of the mirrors and the distances *d* between them. 

It follows that the resolution of a conventional Fabry–Perot interferometer increases linearly with the distance between the mirrors.

If the condition $\Gamma L>\frac{1}{2}\ln\frac{4R}{1-R^{2}}$ is met, the transmission line width of an interferometer based on periodic structures will be less than the line width of conventional interferometers. 

### Influence of Absorption

The results obtained above relate to the case of periodic media without absorption. Absorption can be taken into account if in the resulting Formula (4) a replacement Δk→Δk+iα is made, where $\alpha =2k_{0}n_{i}$ is the absorption coefficient of the light in the periodic structure, $n_{i}$ is the imaginary part of the refractive index of the periodic structure (mirror). 

**Figure 6:**
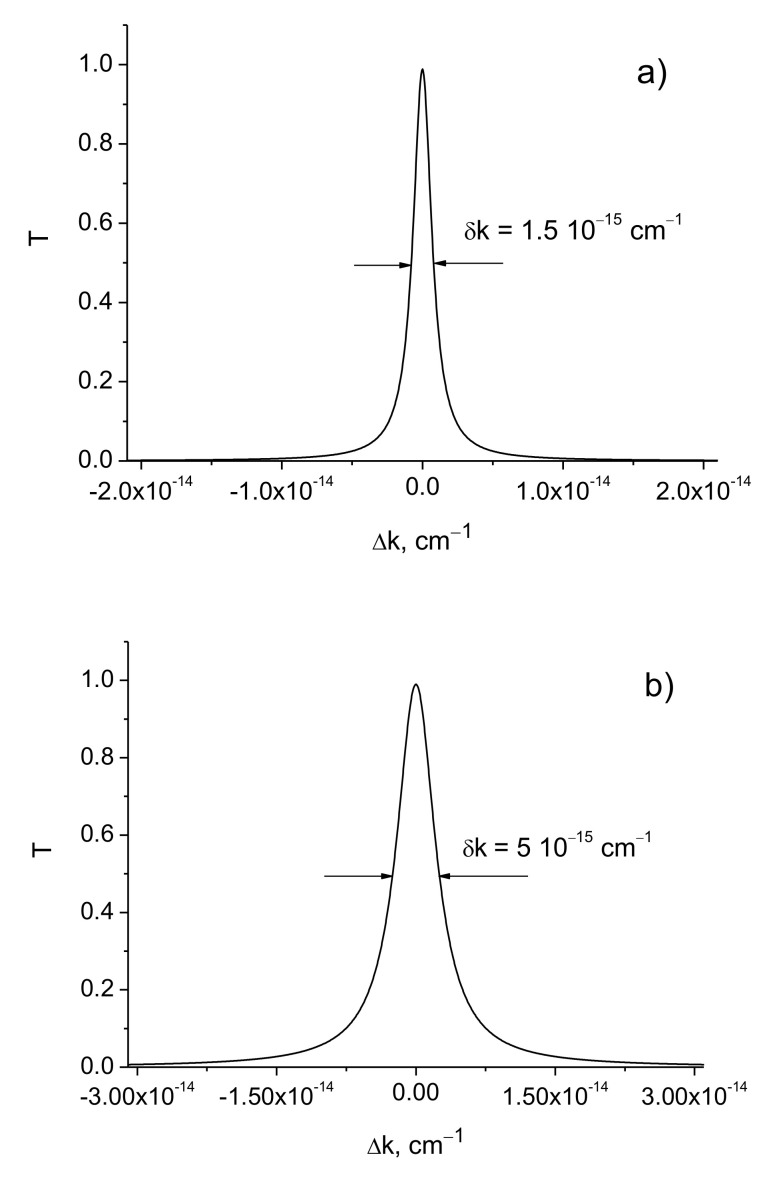
Transmission curves for different values of absorption. (**a**) $n_{i}=1.2\cdot 10^{-8}$; (**b**) $n_{i}=10^{-6}$. $\Delta \varepsilon =2.1\cdot 10^{-4}$; *λ* = 630 nm; *L* = 0.75 cm; *d* = 1 m.

Figure 6 shows the transmission curves for different attenuation values. As the calculations show, the bandwidth increases with increasing absorption (Table 6).

Note that the absorption coefficient in an optical glass BK7 is $\alpha =2.4\cdot 10^{-3}$ cm^−1^, $n_{i}=1.2\cdot 10^{-8}$ and $\alpha =3\cdot 10^{-6}$ cm^−1^ in a medium of SiO_2_ material at a radiation wavelength of λ=0.63 μm [21]. Current technologies permit the manufacture of glasses for optical fiber cores with an absorption coefficient of 10^−7^ cm^−1^.

## 3. Method for Detecting Gravitational Waves 

**Figure 7:**
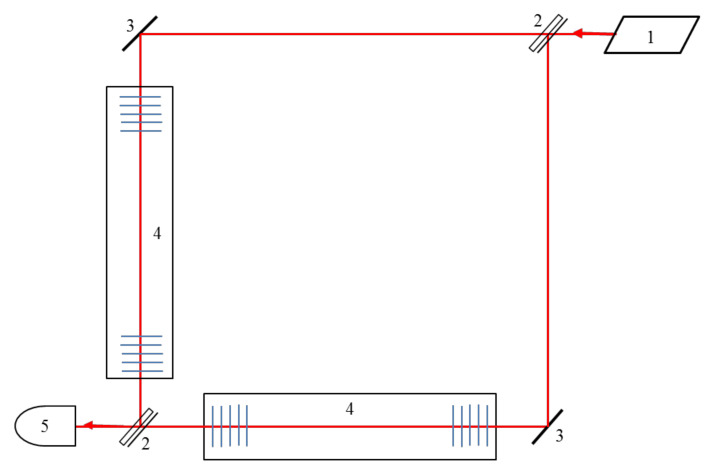
Optical scheme of Mach-Zehnder interferometer with periodical structures as reflecting mirrors. 1—laser, 2—beam splitter, 3—mirror, 4—Fabry-Perot resonator with periodical structures, 5—photodetector.

The experimental installations (the LIGO and Virgo detectors) for detecting gravitational waves are based on a Michelson interferometer with Fabry–Perot resonators in each arm. The distance between the mirrors in the Fabry–Perot resonators changes under the influence of a gravitational wave. Such a laser interferometer as a method for detecting gravitational waves was first proposed in Reference [1]. Similarly, the Mach–Zehnder interferometer can also be used to register gravitational waves (Figure 7).

Under the influence of a gravitational wave, the distance between two free bodies changes [5,6,7]:

(10) $$d\to d_{0}+\Delta d=d_{0}\left(1+h\right),$$

where Δd is the small displacement of mirrors and *h* is the amplitude of a gravitational wave. 

Currently, LIGO antennas have achieved a sensitivity $h≃10^{-21}$ that is sufficient to detect gravitational radiation from the merger of two black holes [2].

When mirrors are shifted, the lines corresponding to non-zero order modes are shifted in the transmission spectrum. The shift of the resonant transmission lines and the change in the distance between the peaks lead to a change in the correlation function measured in the experiment.

The sensitivity of the proposed method can be estimated from the shift of lines in the transmission spectrum when the distance between mirrors changes under the action of a gravitational wave in one of the arms of the Mach–Zehnder interferometer with Fabry–Perot resonators.

To resolve very small offsets, narrow transmission lines are required. Small shifts of wide lines are difficult to distinguish. Narrow lines can be obtained by increasing the distance between mirrors (inversely proportional dependence), or by increasing the value Δε (exponential dependence).

Frequency shifts of peaks of resonant lines are defined by the expression:

(11) $$\Delta \xi_{m}=-\frac{2m\pi \Delta d}{d^{2}},$$

where Δd is the displacement of mirrors.

Thus, the frequency shifts of maxima increase with the mode number m=±1,±2,…,.

The change in distance Δd is related to the frequency shift $\Delta \xi_{m}$ by the relationship

(12) $$\frac{\Delta \xi_{m}}{\xi_{m}}=-\frac{\Delta d}{d}=h≃10^{-21}.$$

The resolution of the interferometer is determined by the width of the transmission line δk. 

Two lines are usually considered resolvable when their maxima are separated by a distance equal to the width of the transmission line:

(13) $$\delta k\leq \Delta \xi_{m}=\xi_{m}h=\frac{2\pi m}{d}h.$$

It follows that for the distance between mirrors *d* = 1 m, the width of the transmission line with the number *m* = 1 must satisfy the condition $\delta k\leq 6.28\cdot 10^{-23}$ cm^−1^.

The main parameters that affect the sensitivity of the interferometer are the amplitude of the change in the dielectric constant Δε, the thickness of the crystal *L*, and the wavelength of the radiation λ.

Changing the distance between mirrors affects the intensity correlation function of intensities

(14) $$F=\langle I(\Delta k,\xi_{m},d)|I(\Delta k,\xi_{m},d+\Delta d)\rangle .$$

As follows from the calculations, high contrast of the interference pattern can be obtained by tuning the interferometer to measure the correlation function of radiation intensities from the two arms of the Mach–Zehnder interferometer corresponding to non-zero resonance modes with m≠0. Note that both the amplitude and intensity correlations of the interferometer output signal can be used to determine the information about the small displacement of the mirrors. Here, in contrast to the amplitude correlation function (interference fringes) for the zero-order mode [14], the correlation function of the intensities corresponding to non-zero-order resonant modes is considered.

**Figure 8:**
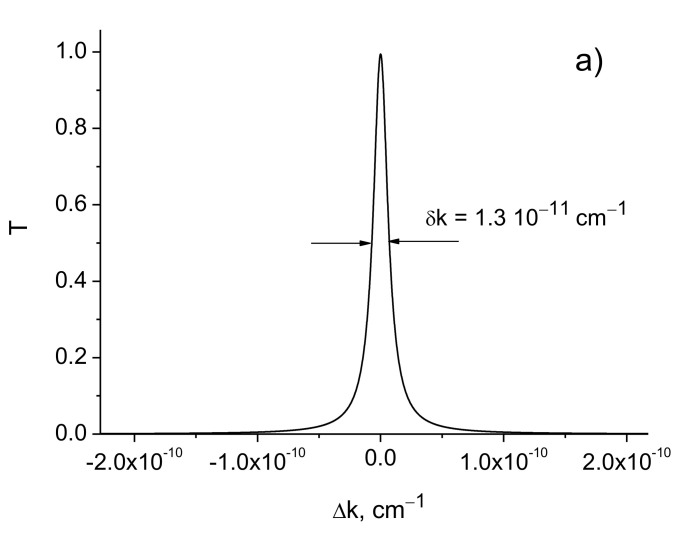

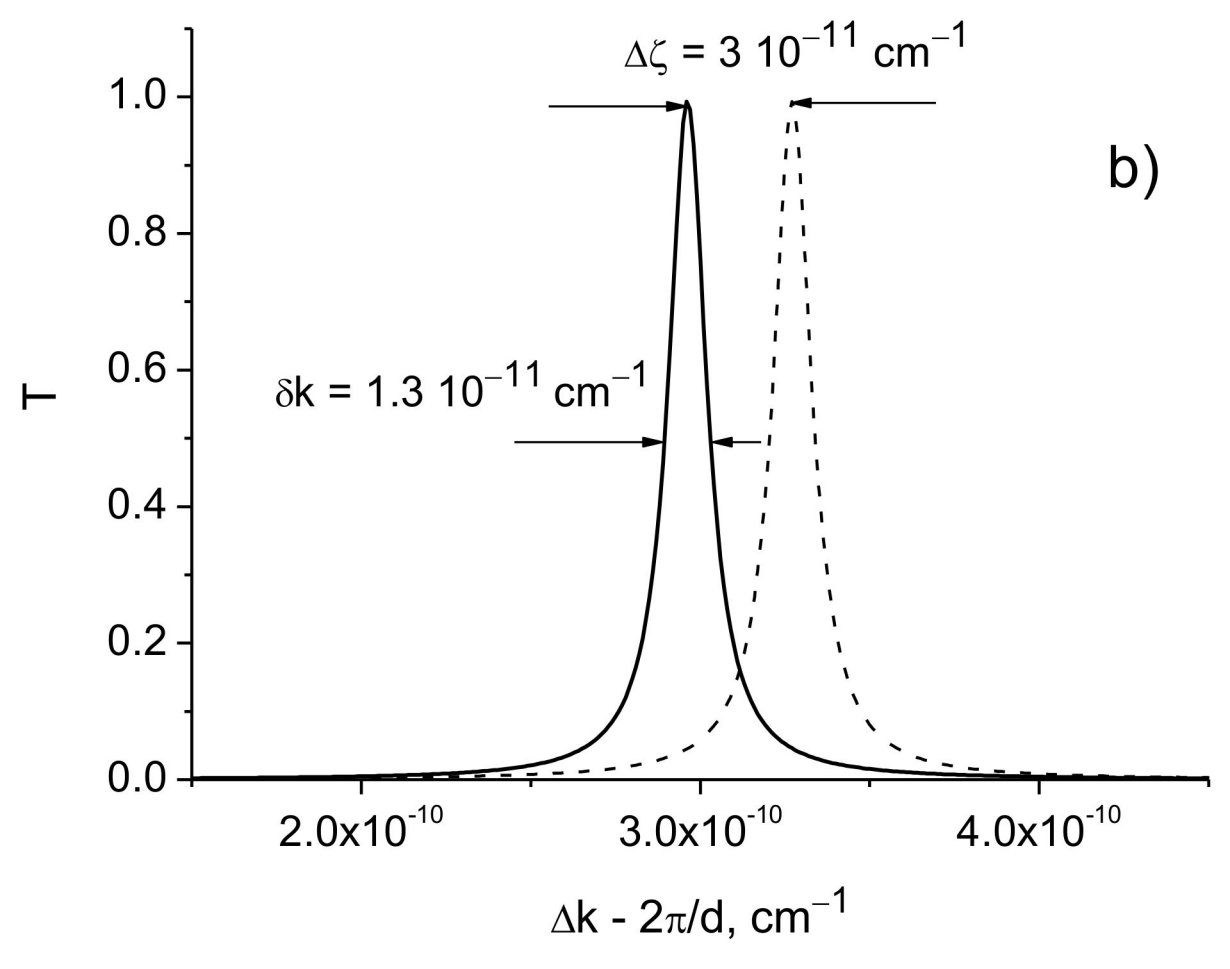
Transmission coefficient as function of the detuning of the resonator: $\Delta \varepsilon =1.5\cdot 10^{-4}$, *λ* = 630 nm, *L* = 0.75 cm, *d* = 1 m, $\Delta d=5\cdot 10^{-8}$ cm. (**a**) *m* = 0; (**b**) *m* = 1, solid curve corresponds to the distance *d* = 1 m, dashed curve corresponds to the distance d+Δd

Figure 8 shows the transmission intensities from the resonator with the distance *d* and from the resonator with the distance d+Δd between the mirrors for the resonant modes of the zero order (Figure 8a) and the first order (Figure 8b).

As follows from the calculations, the transmission lines are resolved when the condition $\delta k\leq \Delta \xi_{m}$ is met, where $\Delta \xi_{m}$ is the shift of the non-zero resonant mode caused by the displacement of the mirror.

**Figure 9:**
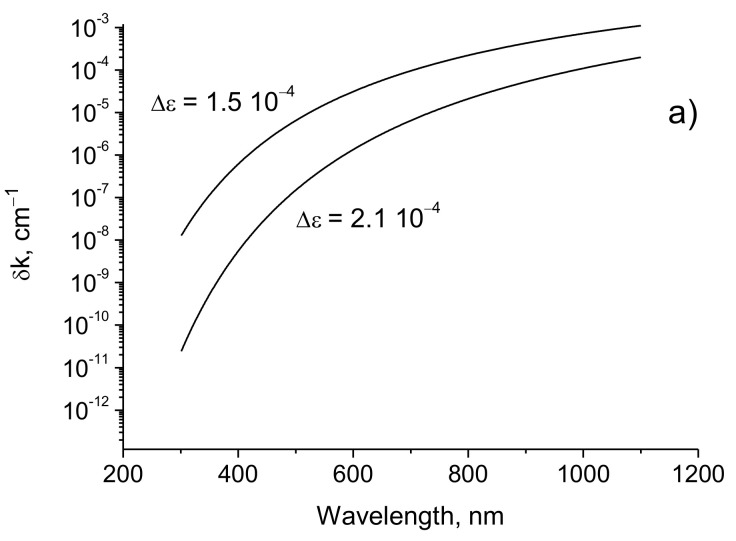

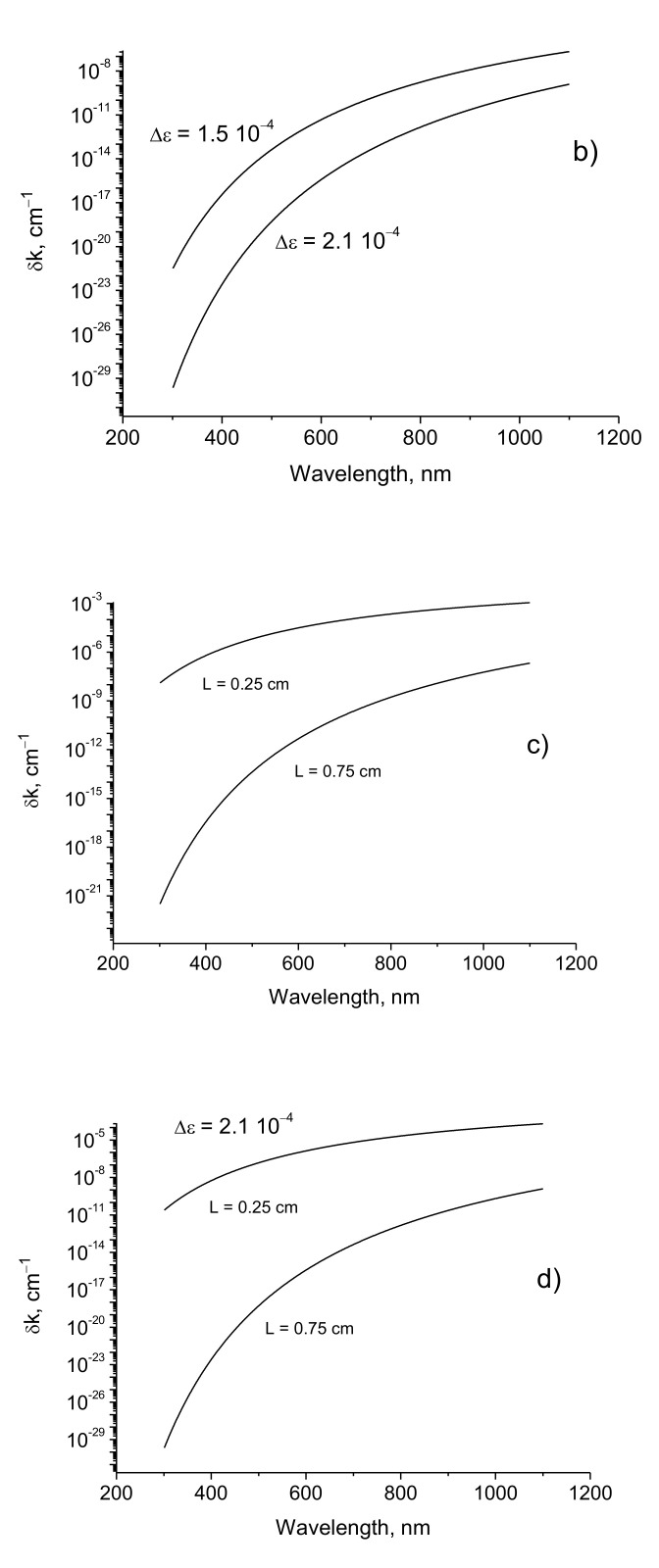
The widths of the transmission spectrum lines as function of radiation wavelength. (**a**) *L* = 0.25 cm; (**b**) *L* = 0.75 cm; (**c**) $\Delta \varepsilon =1.5\cdot 10^{-4}$; (**d**) $\Delta \varepsilon =2.1\cdot 10^{-4}$. *d* = 1 m, *λ* = 630 nm.

The widths of the transmission spectrum lines decrease linearly with the increase of the distance between the periodic structures. The amplitude of the change in the dielectric constant Δε, the thickness of the crystal *L*, and the wavelength of the radiation λ affect the width of the spectral line much more strongly. In Figure 9, the dependences of the spectral line widths on the radiation wavelength are presented for different values of the thickness of the periodic structure and the amplitude of the modulation of the dielectric constant.

It can be seen that the width of the transmission spectrum lines decreases sharply with a decrease in the radiation wavelength and with an increase in the thickness of the periodic structure and the amplitude of the modulation of the dielectric constant.

## 4. Discussion

The results obtained show that the distance between periodic structures can be reduced significantly compared to the distance between mirrors in a LIGO installation. The results demonstrate the possibility of creating an installation for detecting gravitational waves in the laboratory. The use of conventional interferometers for this purpose is currently impossible due to technological difficulties in achieving the required values of the mirror reflection coefficient. Note that the reflectivity of multilayer mirrors used in measurements of gravitational waves is about $(1-R)\sim 10^{-6}$ [7]. It is assumed that technological capabilities will make it possible to achieve values of the order of $(1-R)\sim 10^{-8}$. However, this is not enough to reduce the distance between mirrors to laboratory values. Efforts are constantly being made to increase the sensitivity of the detectors by improving the mechanical properties of the mirror coatings. For LIGO and Virgo installations, a very uniform coating is required on very large surfaces with a diameter of several tens of centimeters. The main objectives of the production are to achieve a large uniform coating while maintaining low optical and mechanical losses. Current mirrors in GW detectors use a stack of silica material (SiO_2_) and titanium-doped tantalum (Ti:Ta_2_O_5_) deposited on a large silica substrate [22]. The optical characterization of these materials by spectroscopic ellipsometry is reported in Reference [23]. Recently, high-reflection dielectric Bragg mirrors were manufactured for the Virgo installation, consisting of two materials: a layer of silica with a low refractive index (*n* = 1.45 at 1064 nm) and titania-doped tantala with a high refractive index (*n* = 2.09) [24]. The required technical characteristics of flatness and roughness are achieved throughout the mirror with a diameter of 300 mm with coatings up to 38 layers with a thickness of 5.9 microns. Although recent improvements in thin-film technology allow for the design and manufacture of narrow-band filters, the requirements for increasing spectral resolution lead to more complex manufacturing. The high stored power in the interferometer leads to thermo-optical distortions from the absorption of optical power, so a complex thermal compensation system must be used in the experiment.

Despite very high-quality optical properties and uniformity, mirror coatings are the dominant source of thermal noise of detectors, limiting the sensitivity of the measurements. While increasing the number of layers will increase the reflectivity of the mirrors, it will also lead to an increase in thermal noise. We suggest using a bulk mirror material to fabricate a volume Bragg grating (VBG), which will allow better heat dissipation and increase the reflectivity of mirrors and resolution of the interferometer. Such VBGs can be recorded by holographic methods inside photothermorefractive glasses.

The interferometer considered indicates the possibility of creating a laboratory installation for measuring gravitational waves. The Fabry–Perot resonator with periodic structures as reflecting mirrors has an ultra-narrow bandwidth of laser radiation. Note that in contrast to LIGO, in the proposed system, the distributed mirrors work not on reflection, but on the transmission of the incident light. In addition, the design with photonic crystals (sinusoidal periodic structures) facilitates the cooling of mirrors. 

The resonant transmission of radiation through the periodic structure has a simple explanation. It is known from quantum mechanics that if a free particle has an energy that coincides with the energy of the quantum level between the barriers, then the particle passes through such barriers. The case under consideration is essentially a classical analog of such resonant tunneling. 

Resonant phenomena during wave propagation in inhomogeneous plane-layered media lead to a steep increase in the transmission of waves with a certain wavelength. In quantum mechanics, a similar effect is observed for de Broglie waves resonantly passing through a system of two potential barriers (the Ramsauer effect). Resonant FTIR (frustrated total internal reflection) filters consisting of layered media are widely known in optics [25]. The bandwidth of such filters based on existing optical materials in the visible wavelength range is on the order of several nanometers [26].

The considered resonators with periodic structures, in contrast to the conventional Fabry–Perot interferometer, have an exponentially narrow bandwidth of laser radiation. As follows from the calculations, the line widths in the transmission spectrum of the structures under consideration decrease drastically with an increase in the amplitude of the dielectric constant modulation Δε and an increase in the thickness of the periodic structure *L*. Therefore, a significant increase in the resolution can be reached if the modulation amplitude or the thickness of the periodic structure is increased. Besides, the resolution of the interferometer increases significantly with a decrease in the wavelength of the laser radiation. The maximum resolution values are limited by the presence of light absorption in the crystals. However, high-purity materials with low absorption coefficients are available at present. For example, the absorption coefficients of glasses in the optical fiber cores are only of the order of 10^−7^ cm^−1^. 

To achieve the necessary sensitivity of the interferometer for the detection of gravitational waves, large amplitudes of the dielectric constant modulation are required. Current technologies allow the production of periodic structures with the parameters considered above. In acousto-optic crystals, it is possible to create tunable modulation of the refractive index with the help of ultrasound. Low-loss high-efficiency volume Bragg gratings (VBG) in glasses can be recorded by holographic methods. In References [27,28], the designed volume Bragg grating was fabricated inside photothermorefractive (PTR) glasses. The refractive index modulation (RIM) values inside the VBG recorded in PTR glasses were $\Delta n=4.78\cdot 10^{-4}$ $\left(\Delta \varepsilon ≃1.4\cdot 10^{-3}\right)$ and $\Delta n=4.37\cdot 10^{-4}$ $\left(\Delta \varepsilon ≃1.3\cdot 10^{-3}\right)$ at wavelengths 632.8 nm and 1064 nm, accordingly [28]. These values of the RIM are sufficient to achieve the required sensitivity of the interferometer for detecting gravitational waves. A compact detector for detecting gravitational waves is highly desirable. Recently a compact detector for space–time metric and curvature was considered [29]. It was shown that quantum spatial superpositions of mesoscopic objects could be exploited to create such a detector. Such detectors can be used also for detecting extremely weak signals such as mid-frequency and low-frequency GWs. This device will not replace but will supplement the existing installations. The fact is that LIGO and Virgo only accept high-frequency gravitational waves: from tens to thousands of hertz. At the same time, the new device will be sensitive to waves in the range from a millionth of a hertz to ten hertz [29]. To register waves of such frequencies by the usual method (like LIGO and Virgo), detectors hundreds of thousands of kilometers in size would be required.

In Reference [30], a three-dimensional gravitational wave detector with three Michelson interferometers setting in a regular triangular pyramid, which has a more spherically symmetric antenna pattern, is proposed. 

More recently, radio telescopes have been proposed to search for GW in a wide frequency range [31]. The fact is that gravitational waves are converted into photons and vice versa in the presence of magnetic fields. The distortion of the cosmic microwave background caused by this transformation can serve as a detector of gravitational wave sources from MHz to GHz. 

## 5. Conclusions

Thus, using Fabry–Perot resonators with periodic structures as reflecting mirrors can significantly reduce the size of the system (the distance between the mirrors can be only a few meters). The method based on the measurement of the correlation function of the intensities of non-zero-order resonant modes from the two arms of the Mach–Zehnder interferometer allows obtaining a resolution sufficient for the registration of gravitational waves. The resolution of the interferometer of the order of $\delta k≃10^{-23}$ cm^−1^ at the distance between the mirrors *d* = 1 m can be obtained for practically achievable parameters of the periodic structure. It is shown that a significant increase in the sensitivity and a decrease in the size of the detector are also possible with a decrease in the wavelength of the radiation source. The sensitivity of such an interferometer for small movements of reflecting structures relative to each other exceeds the sensitivity of the LIGO system with practically achievable parameters of the periodic diffraction structures.

## Figures and Tables

**Table 1:** The widths of transmission curves δk for different modulation amplitudes Δε and wavelengths λ . *L* = 0.75 cm, *d* = 1 m.

Δε	λ, nm	$\delta k,\;{\mathrm{cm}}^{-1}$
$2.1\cdot 10^{-4}$	630 1064	$1.5\cdot 10^{-15}$ $6\cdot 10^{-10}$
$2.9\cdot 10^{-4}$	630 1064	$7.8\cdot 10^{-21}$ $5.4\cdot 10^{-13}$

**Table 2:** The widths of transmission curves δk amplitudes of transmission coefficients *T*_max_ for different modulation amplitudes Δε and asymmetry values δL. *L* = 0.75 cm, *d* = 1 m, *λ* = 630 nm.

Δε	δL, μm	$\delta k,\;{\mathrm{cm}}^{-1}$	*T*_max_
$2.1\cdot 10^{-4}$	10 100	$1.6\cdot 10^{-15}$ $1.4\cdot 10^{-15}$	0.997 0.97
$2.9\cdot 10^{-4}$	5 10	$2.3\cdot 10^{-20}$ $2.6\cdot 10^{-20}$	0.71 0.64

**Table 3:** The widths of transmission curves δk and amplitudes of transmission coefficients *T*_max_ for different modulation amplitudes $\Delta \varepsilon_{2}$ and asymmetry values δε. *L* = 0.75 cm, *d* = 1 m, λ = 630 nm.

$\Delta \varepsilon_{2}$	δε	$\delta k,\;{\mathrm{cm}}^{-1}$	*T*_max_
$2.1\cdot 10^{-4}$	$2.1\cdot 10^{-6}$ $-2.1\cdot 10^{-6}$	$1.4\cdot 10^{-15}$ $1.0\cdot 10^{-15}$	0.98 0.96
$2.9\cdot 10^{-4}$	$8.7\cdot 10^{-6}$ $-8.7\cdot 10^{-6}$	$2.6\cdot 10^{-20}$ $4.0\cdot 10^{-20}$	0.67 0.47

Note that there is an asymmetry with respect to the sign of δε , i.e., different resolutions can be obtained depending on the sign of the difference in the values of the modulation amplitudes in the left and right periodic structures.

**Table 4:** The widths of transmission curves δk for different modulation amplitudes Δε and distances *d. L* = 0.40 cm, λ = 630 nm.

Δε	d, m	$\delta k,\;{\mathrm{cm}}^{-1}$
$3.0\cdot 10^{-4}$	1 3	$3.0\cdot 10^{-12}$ $1.0\cdot 10^{-12}$
$5.0\cdot 10^{-4}$	1 3	$3.3\cdot 10^{-19}$ $1.1\cdot 10^{-19}$

**Table 5:** The widths of transmission curves δk for different values 1 − *R* and distances *d.* λ = 630 nm.

1−R	d, km	$\delta k,\;{\mathrm{cm}}^{-1}$
$6.0\cdot 10^{-6}$	1 4	$2.4\cdot 10^{-10}$ $6.2\cdot 10^{-11}$
$1.0\cdot 10^{-8}$	1 4	$4.0\cdot 10^{-13}$ $1.0\cdot 10^{-13}$

**Table 6:** The widths of transmission curves δk for different modulation amplitudes Δε and absorptions. *λ* = 630 nm, *L* = 0.75 cm, *d* = 1 m.

Δε	$n_{i}$	$\delta k,\;{\mathrm{cm}}^{-1}$
$2.1\cdot 10^{-4}$	$1.2\cdot 10^{-8}$ $10^{-6}$	$1.6\cdot 10^{-15}$ $5.0\cdot 10^{-15}$

## Data Availability

Not applicable.
